# Pregnancy-Associated Vulval Lymphangiectasia With a Non-Vesicular Wart-Like Appearance

**DOI:** 10.14740/jmc5391

**Published:** 2026-10-02

**Authors:** Suppachai Lawanaskol, Kanokkan Suwan, Suphakit Khutanthong

**Affiliations:** a Chaiprakarn Hospital, Chiang Mai, Thailand; b Department of Pathology, Faculty of Medicine, Naresuan University, Phitsanulok, Thailand

**Keywords:** Lymphatic system, Pregnancy, Lymphangiectasia, Genital wart

## Abstract

Vulval lymphangiectasia is a rare benign lymphatic disorder characterized by dilatation of superficial lymphatic vessels of the vulva. Although classically described as clusters of translucent vesicles with a “frogspawn” appearance, it may also present as non-vesicular, skin-colored, or wart-like lesions that can mimic condyloma acuminata and other vulval disorders. Recognition of this morphological spectrum is particularly important during pregnancy, when diagnostic uncertainty may affect counselling and delivery planning. We report a 37-year-old woman with recurrent pregnancy-associated, non-vesicular, wart-like vulval lymphangiectasia that developed during late pregnancy, was confirmed histopathologically, and regressed spontaneously after vaginal delivery. This case reinforces the recognized morphological spectrum of pregnancy-associated vulval lymphangiectasia and highlights the importance of considering this diagnosis when non-vesicular vulval lesions arise during pregnancy.

## Introduction

Pregnancy-induced vulval lymphangiectasia is an uncommon pregnancy-related dermatologic condition characterized by dilatation of superficial lymphatic channels in the vulval skin. Although benign, its clinical appearance can mimic sexually transmitted infections—particularly condyloma acuminata and herpes simplex—leading to unnecessary anxiety, stigma, inappropriate treatment, and potentially inappropriate selection of the route of delivery. The condition also raises practical obstetric concerns, including the risk of lesion trauma during childbirth, bleeding, secondary infection, and uncertainty regarding the safest mode of delivery [1].

The existing literature suggests at least two clinical scenarios with apparently distinct morphological patterns. One presentation is the classic vesicular “frog-egg” or translucent papulovesicular appearance, which is more readily recognized as a lymphatic lesion [2]. In contrast, another presentation is predominantly non-vesicular and may appear verrucous or wart-like, increasing the likelihood of misdiagnosis and overtreatment [2, 3]. Given the limited number of published cases, management is not standardized and typically depends on careful clinical assessment, histopathological confirmation when indicated, and individualized counselling [3].

Here, we present a case of recurrent pregnancy-associated vulval lymphangiectasia that developed during late pregnancy and regressed spontaneously postpartum. This case reinforces the recognized morphological spectrum of vulval lymphangiectasia, particularly its non-vesicular, wart-like presentation, which may be clinically mistaken for condyloma acuminata or other vulval disorders. Recognition of this presentation may therefore be important for avoiding misdiagnosis and for informing counselling and delivery planning during pregnancy.

## Case Report

### Investigations

A 37-year-old woman (gravida 5, para 3 (G5P3013)), with one previous termination of pregnancy, presented at approximately 40 weeks of gestation with multiple papules on both labia majora noted during a routine antenatal visit. She had no pre-existing medical conditions and no history of surgery, radiotherapy, malignancy, trauma, urologic disease, tuberculosis, or sexually transmitted infections. She reported similar lesions during her previous pregnancies but had not sought hospital care at that time; therefore, no medical records were available. She stated that the lesions had previously appeared at around the seventh month of gestation and regressed spontaneously within 1 month after delivery without treatment. In the current pregnancy, she attended antenatal care according to the Thai national standard follow-up program. Screening for pregnancy-related conditions and initial serologic tests were negative. During routine follow-up visits, standard obstetric examinations were performed; however, a per-vaginal (vulval) examination was not routinely undertaken. Therefore, skin changes developing between the initial antenatal visit and the final visit before labor may not have been fully evaluated. The attending physician assessed the vulval skin lesions only when the patient presented in labor. This examination was performed to determine whether the lesions might contraindicate vaginal delivery or complicate intrapartum management, including assessment for cephalopelvic disproportion, rupture of membranes, and the need for urinary catheterization. Vulval lymphangiectasia was not initially suspected and was considered only after the lesions were noted incidentally at presentation in labor. A focused history was then obtained, during which the patient reported that similar lesions had recurred in previous pregnancies and resolved spontaneously after delivery.

On examination, her vital signs were within normal limits. There was no pitting edema, hemorrhoids, superficial thrombosed veins, or lymphadenopathy. Vulval examination revealed multiple painless, non-pruritic nodules measuring 0.5–1.0 cm involving the labia majora and minora bilaterally. The lesions were skin-colored, soft, verrucous in appearance, mobile, and non-vesicular, and were diffusely distributed but confined to the vulva with bilateral symmetry (Fig. 1a). No swelling or signs of infection or inflammation were present. No formal dermatology consultation or dermoscopic examination was performed before histopathological confirmation. The differential diagnosis included condyloma acuminata, herpes simplex infection, angiokeratoma, vulval varicosities or hemangioma, fibroepithelial polyps, and vulval lymphangiectasia.

**Figure 1:**
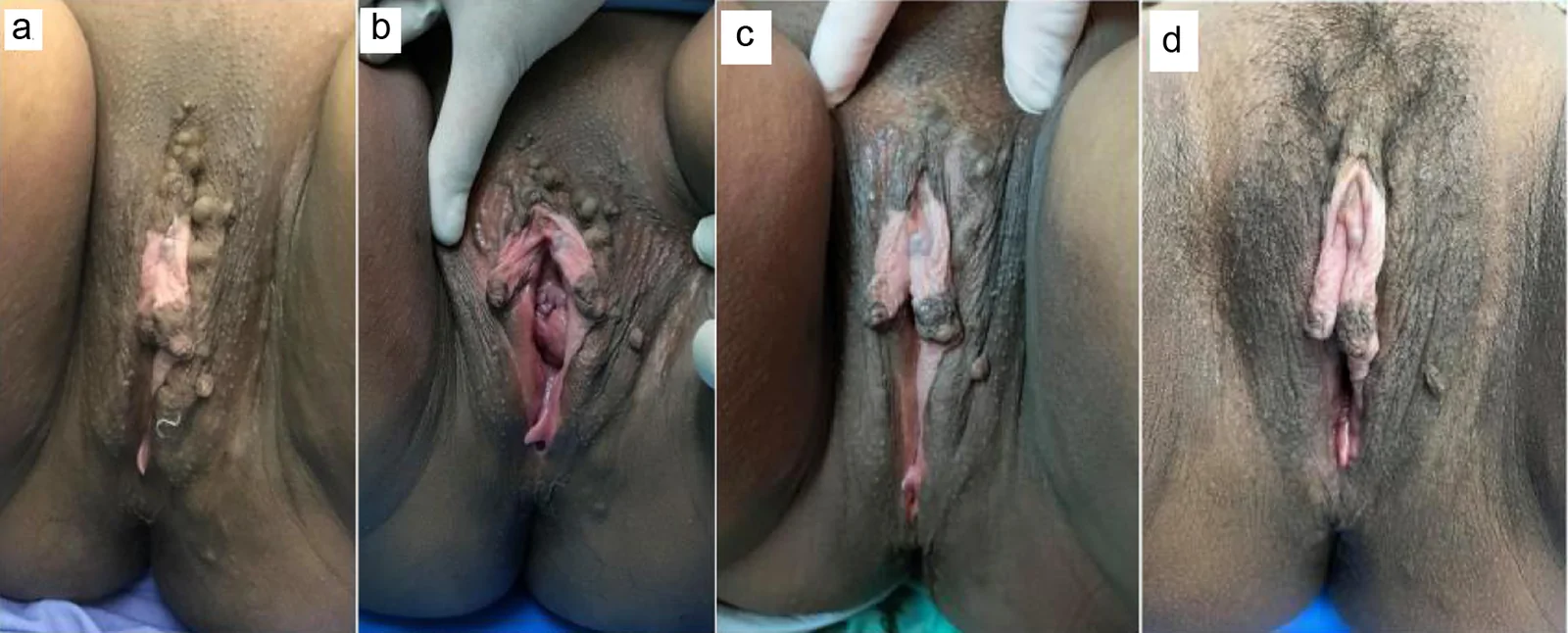
Serial vulval examinations showing multiple skin-colored papules on both labia before delivery (a) and 1 day postpartum (b). The lesions began to regress by 2 days postpartum (c) and had almost completely regressed by 4 weeks postpartum (d).

Human immunodeficiency virus (HIV) testing, syphilis serology, hepatitis B surface antigen (HBsAg), anti-hepatitis C virus (anti-HCV), and a conventional Pap smear (Papanicolaou test) were performed to investigate the cause of the lesion. All tests were negative. She delivered a healthy infant at 40 weeks of gestation (birth weight 3,190 g) via uncomplicated vaginal delivery, with only a mild perineal tear and no postpartum hemorrhage or episiotomy-related complications. Two days after delivery, the lesions began to regress, decreasing in size and number (Fig. 1b, c). By 3 weeks postpartum, most lesions had almost completely regressed, leaving a single nodule on the labia majora (Fig. 1d). An excisional biopsy of the residual lesion was performed to confirm the diagnosis while minimizing disturbance during pregnancy and reducing the risk of bleeding. Histopathological examination showed several dilated, thin-walled, endothelial-lined spaces extending throughout the papillary and reticular dermis (Fig. 2). Additional testing for latent tuberculosis (tuberculin skin test and interferon-gamma release assay) was negative.

**Figure 2:**
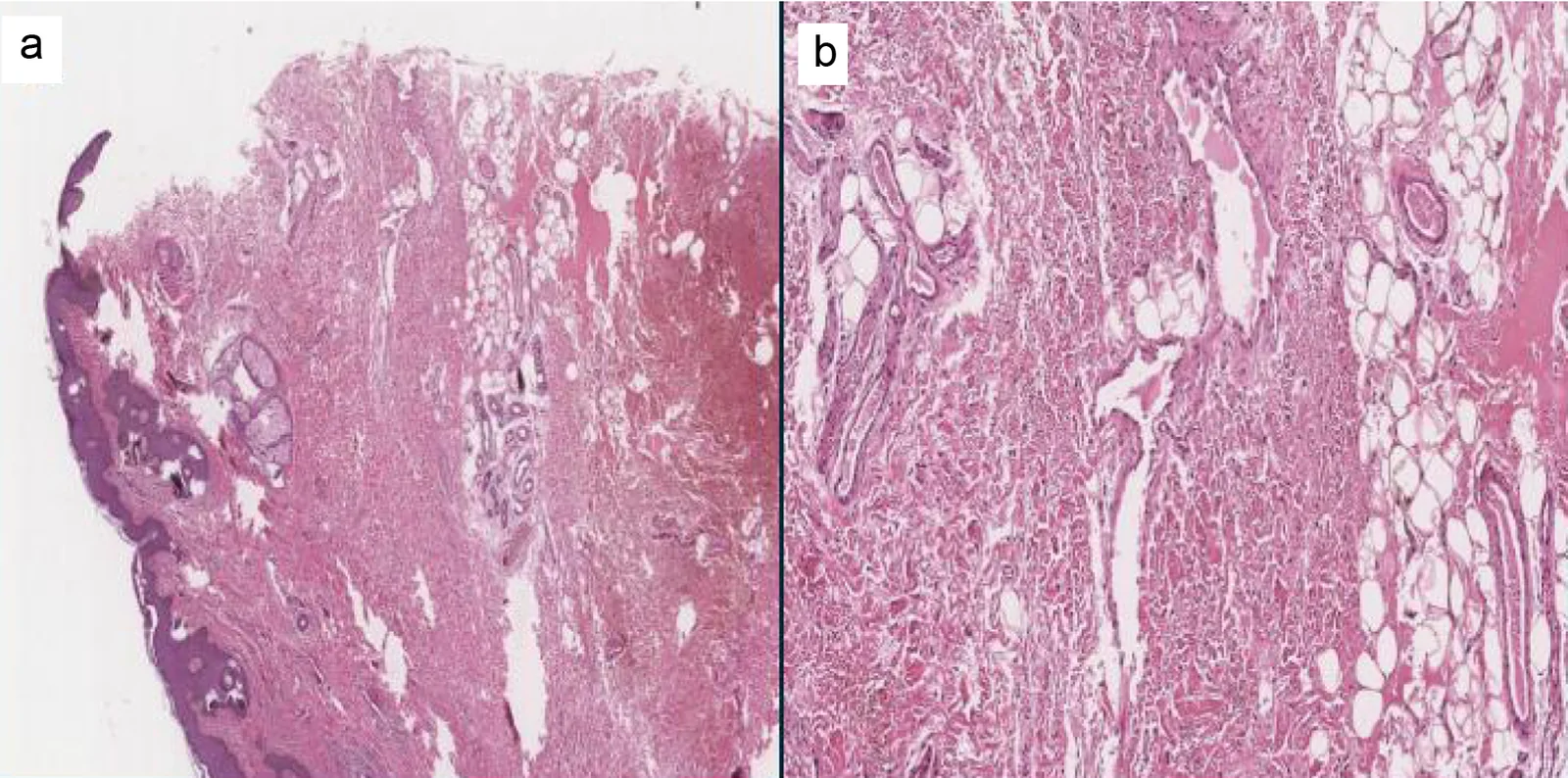
Histopathological findings of the vulval lesion. (a) Hematoxylin and eosin staining showing dilated, thin-walled vascular space within the superficial dermis (original magnification, × 20). (b) Higher-magnification view showing the dilated, thin-walled vascular space lined by a single layer of flattened endothelial cells (original magnification, × 40).

### Diagnosis

We divided the diagnostic process into two components: (1) antepartum/intrapartum considerations and (2) postpartum considerations.

For the antepartum and intrapartum assessment, we obtained a history of lesion characteristics in previous pregnancies based on the patient’s verbal description; however, this approach may be imprecise and can lead to diagnostic uncertainty.

For the postpartum assessment, we recommended close follow-up and, when indicated, excisional biopsy with submission of tissue for histopathologic evaluation and immunohistochemical staining.

Furthermore, during follow-up, the patient’s pubic hair—routinely shaved to prepare for potential emergency caesarean section (e.g., cephalopelvic disproportion or non-reassuring fetal status)—was shaved intrapartum, immediately before delivery. Subsequent natural regrowth of pubic hair facilitated serial clinical assessment and supported spontaneous regression of the lesions (Fig. 1b–d).

### Treatment

Management was divided into two components: obstetric considerations and lesion-specific considerations. From an obstetric perspective, because misdiagnosis is a potential concern, counselling regarding the option of cesarean delivery may be appropriate—particularly when an infectious etiology cannot be confidently excluded and the patient prefers vaginal delivery. From a lesion-specific perspective, serial documentation (ideally standardized clinical photographs) can facilitate day-by-day or week-by-week comparison and help assess gradual regression or progression of the lesion.

### Follow-up and outcomes

The patient was followed for 4 weeks postpartum, during which the lesions regressed completely. No recurrence was observed over 1 year of follow-up. No further imaging was performed because the lesions resolved spontaneously; ultrasonography was considered unlikely to add diagnostic value, and computed tomography or magnetic resonance imaging was not pursued to avoid unnecessary exposure to radiation and contrast media. The patient provided informed consent for publication of de-identified clinical photographs of the vulva.

## Discussion

We report a recurrent pregnancy-associated vulval lymphangiectasia presenting predominantly as non-vesicular, skin-colored, wart-like papules rather than the classic translucent “frogspawn” appearance. The lesions developed during late pregnancy, recurred in a similar pattern across previous pregnancies, and regressed spontaneously after delivery. Histopathological examination with lymphatic staining confirmed the diagnosis. The principal clinical significance of this presentation lies in its potential to mimic condyloma acuminata and other vulval disorders, thereby creating diagnostic uncertainty and potentially influencing counselling and delivery planning.

Lymphangiectasia refers to dilatation of lymphatic vessels, usually secondary to lymphatic damage or obstruction, and has been associated with radiotherapy, surgery, trauma, malignancy, tuberculosis, sexually transmitted infections, filariasis, and pregnancy. Vulval lymphangiectasia typically presents as vesicles or papules, sometimes with serosanguineous leakage, and the classic “frogspawn” appearance describes clustered translucent vesicles. In contrast, the present case showed non-vesicular, normopigmented papules with an intact epidermal surface. Histopathology typically confirms the diagnosis by demonstrating dilated lymphatic spaces lined by flattened endothelial cells within the dermis [1, 4].

The clinical course supports pregnancy as a likely precipitating factor, although the precise pathophysiological mechanism remains uncertain. Acquired lymphangiectasia is thought to result from impaired lymphatic drainage, leading to increased intralymphatic pressure, dermal backflow, and subsequent dilatation of superficial dermal lymphatic vessels. During pregnancy, mechanical compression of pelvic lymphatic and venous outflow by the gravid uterus, together with increased venous stasis, weight gain, and pregnancy-related physiological and hormonal changes, may further compromise lymphatic drainage. Previous reports have similarly proposed pregnancy-related impairment of lymphatic circulation as a mechanism for the development or enlargement of vulval lymphatic lesions. In our patient, lesions repeatedly developed during late pregnancy and regressed spontaneously after delivery, including complete regression by the fourth postpartum week in the current pregnancy. This reproducible temporal pattern supports a pregnancy-associated process, although it does not establish a specific causal mechanism [1, 3, 5–8].

A definitive diagnosis of vulval lymphangiectasia cannot always be made clinically, particularly when the classic translucent “frogspawn” appearance is absent. As in the case described by Verma, our patient had skin-colored, non-vesicular lesions, making differentiation from condyloma acuminata and other sexually transmitted or neoplastic lesions difficult. Clinical distinction from other vulval lesions may be difficult when lymphangiectasia lacks its classic translucent vesicular appearance. Condyloma acuminata typically presents as verrucous or papillomatous lesions and may closely resemble the wart-like morphology seen in the present case, although the recurrent association with pregnancy and spontaneous postpartum regression may provide an important clinical clue against an infectious etiology. Herpes simplex infection more commonly produces painful grouped vesicles or erosions, whereas vulval lymphangiectasia is often painless and may remain intact without ulceration. Angiokeratomas are usually red to violaceous vascular papules and may bleed after minor trauma, while vulval varicosities generally appear as compressible, bluish, dilated venous structures. Fibroepithelial polyps are benign polypoid stromal lesions that may enlarge during pregnancy but are typically solitary or fewer in number, pedunculated, and do not characteristically show the clustered superficial lymphatic morphology of lymphangiectasia [9]. Nevertheless, substantial morphological overlap exists among these conditions, and histopathological examination is important when the clinical diagnosis remains uncertain.

A recent report by Fawzy et al further illustrates the clinical heterogeneity of pregnancy-associated vulvar lymphatic disorders [2]. Their patient developed a giant unilateral vulvar lymphangioma circumscriptum during pregnancy in association with ipsilateral primary lower-limb lymphedema; notably, the lesion was initially misdiagnosed clinically as condyloma acuminata and histologically as a fibroepithelial polyp. In contrast, our patient had bilateral, predominantly non-vesicular wart-like lesions without lower-limb lymphedema or other evident pre-existing lymphatic obstruction, and the lesions showed a reproducible pattern of occurrence during pregnancy followed by spontaneous postpartum regression. Together, these cases highlight the variable morphology and underlying clinical context of pregnancy-associated vulvar lymphatic lesions and emphasize the potential for diagnostic confusion with both condylomatous and benign polypoid lesions.

Accurate diagnosis is important for counselling, management, and delivery planning. Although condyloma acuminatum was initially the main differential diagnosis and cesarean delivery was discussed, the patient preferred vaginal delivery because of similar uneventful lesions in previous pregnancies. Postpartum biopsy confirmed the diagnosis. Unlike some reported cases associated with lymphatic obstruction, our patient had no pitting edema or lower-limb involvement [2, 10, 11].

A limitation of this report is that dermoscopic examination was not performed before biopsy. Dermoscopy might have provided additional morphological information and assisted in the clinical differential diagnosis; however, definitive diagnosis in this case was established histopathologically with supportive lymphatic structure staining.

The timing of biopsy should be individualized. Although antenatal biopsy has been reported, our patient presented late in pregnancy, and biopsy was therefore deferred until postpartum to reduce procedural risk and potential bleeding. Because vulval lymphangiectasia may coexist with conditions causing lymphatic obstruction, selected investigations may be appropriate. In this case, however, the asymptomatic course and spontaneous postpartum regression supported conservative management and allowed further documentation of the recognized non-vesicular clinical presentation [3, 12]. All serological testing was negative.

Management should be individualized according to lesion morphology, diagnostic uncertainty, gestational age, patient preference, and obstetric indications. Observation with close postpartum follow-up is often appropriate, particularly for asymptomatic lesions that regress spontaneously. Biopsy may be considered when lesions are persistent, enlarging, symptomatic, clinically uncertain, or when histopathological confirmation would change management. In urgent intrapartum situations, visual inspection alone may be insufficient to distinguish infectious from noninfectious lesions; therefore, delivery planning should consider the overall obstetric context and the likelihood of transmissible infection. In non-emergent cases, histopathological and/or immunohistochemical confirmation can be coordinated with the anticipated delivery timeline. Extensive surgical or ablative treatment is generally unnecessary when lesions are asymptomatic and show spontaneous postpartum regression.

The morphology of our patient’s lesions was most similar to the early pregnancy-related case described by Verma, showing a predominantly non-vesicular, papillomatous, or wart-like appearance rather than the classic translucent “frogspawn” pattern [3]. Recognition of this phenotype is clinically important because it may mimic condyloma acuminata or herpetic infection, conditions that can substantially affect counselling and delivery planning.

### Learning points

Pregnancy-induced vulval lymphangiectasia may present as non-vesicular, skin-colored, wart-like papules rather than the classic translucent “frogspawn” appearance, and may closely mimic condyloma acuminata or other infectious vulval lesions.

Recurrent lesions during pregnancy with spontaneous postpartum regression are an important clinical clue suggesting a pregnancy-related lymphatic process, although clinical assessment alone may be insufficient for definitive diagnosis.

Histopathological examination with lymphatic immunohistochemistry can confirm the diagnosis and help avoid misdiagnosis, overtreatment, and unnecessary concern regarding sexually transmitted infection or malignancy.

Delivery planning should be individualized according to obstetric indications, lesion morphology, diagnostic uncertainty, and patient preference. In asymptomatic lesions that regress postpartum, conservative management with close follow-up may be appropriate.

## Data Availability

Due to patient privacy and the identifiable nature of clinical images, the full dataset of additional clinical photographs and histopathology images (including slide block and special-stain images) is not publicly available. These materials may be shared by the corresponding author upon reasonable request and with appropriate safeguards for confidentiality.
